# Supplementary material for: Spectral graph model for fMRI: A biophysical, connectivity-based generative model for the analysis of frequency-resolved resting-state fMRI
Source: Imaging Neurosci (Camb). 2024 Dec 9;2:imag-2-00381. doi: 10.1162/imag_a_00381 (PMC12315728; doi:10.1162/imag_a_00381)
Supplement: Supplementary Material [file imag_a_00381-supp.pdf]

Spectral graph model for fMRI: a biophysical, connectivity-based generative model for the analysis of frequency-resolved resting state fMRI

Ashish Raj
Department of Radiology and Biomedical Imaging, and
Graduate Program in Bio-engineering
University of California, San Francisco
San Francisco, CA 94143
ashish.raj@ucsf.edu

Benjamin S Sipes
Department of Radiology and Biomedical Imaging, and
Graduate Program in Bio-engineering
University of California, San Francisco
San Francisco, CA 94143
benjamin.sipes@ucsf.edu

Parul Verma
Department of Radiology and Biomedical Imaging
University of California, San Francisco
San Francisco, CA 94143
parul.verma@ucsf.edu

Daniel H. Mathalon
Department of Psychiatry and Behavioral Sciences, UCSF
University of California, San Francisco, and
Veterans Affairs San Francisco Health Care System
San Francisco, CA 94121
daniel.mathalon@ucsf.edu

Bharat Biswal
Department of Biomedical Engineering
New Jersey Institute of Technology
619 Fenster Hall, Newark, NJ 07102
biswal@njit.edu

Srikantan Nagarajan
Department of Radiology and Biomedical Imaging, and
Graduate Program in Bio-engineering
University of California, San Francisco
San Francisco, CA 94143
srikantan.nagarajan@ucsf.edu

August 23, 2024

Supplementary Material

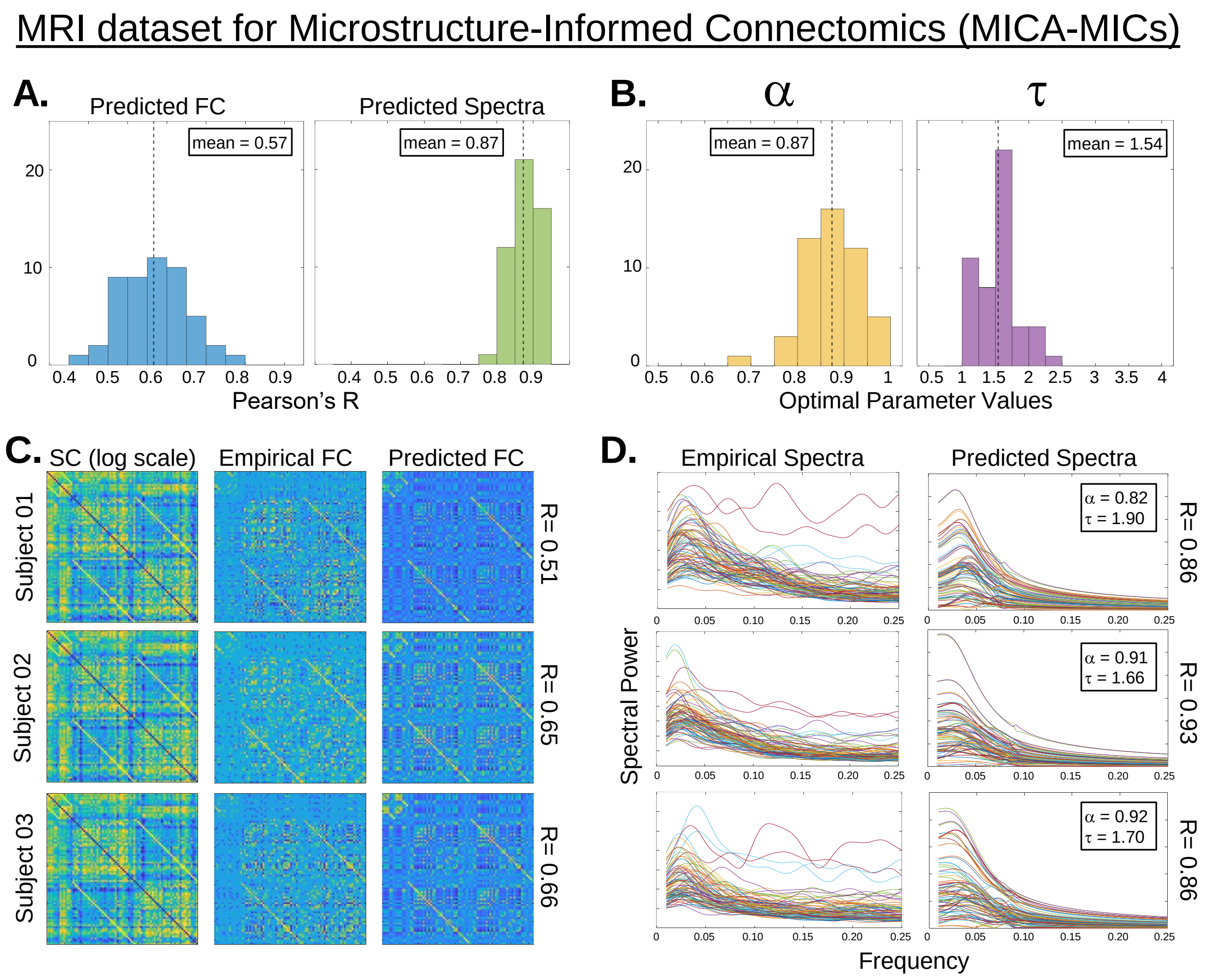

Figure S1: **Replication with the high bandwidth public MICA fMRI dataset** ($N = 50$), analogous to Figure 3. The SGM model performed equally well in reproducing the faster and higher bandwidth MICA data ($TR = 0.6$ sec). Compared to the main UCSF study results, the histograms in panel A of goodness of fit (Pearson's R) are similar, although the fitted model parameters in panel B are more narrowly-distributed.

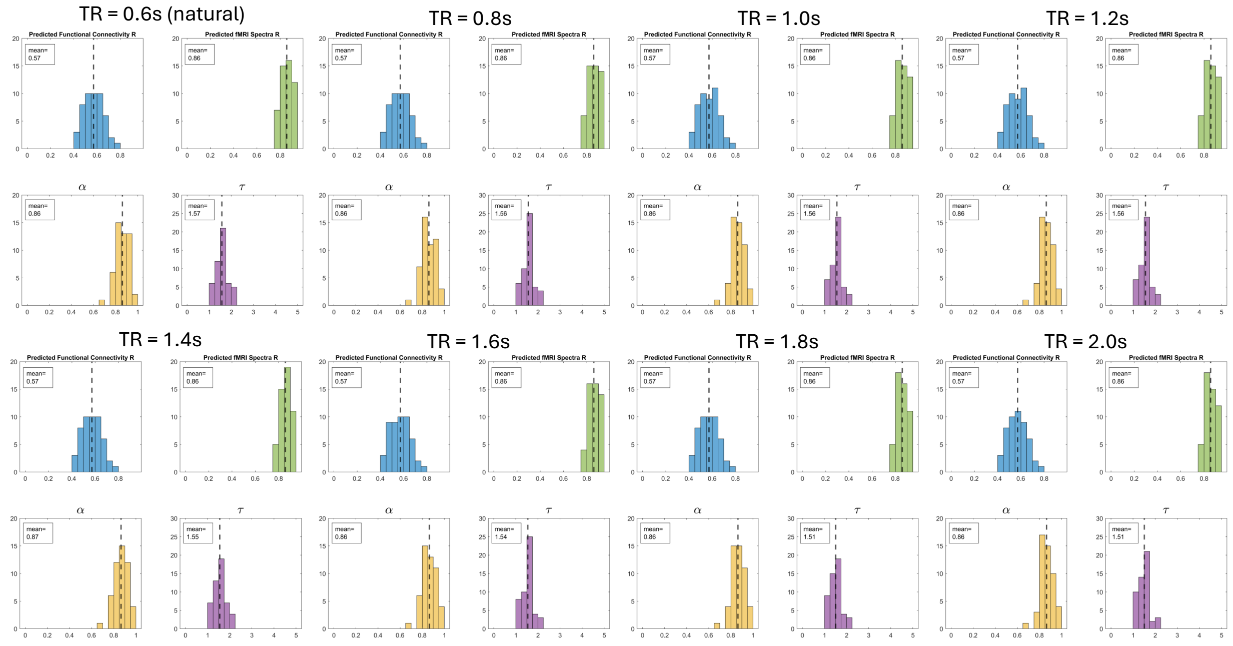

Figure S2: **SGM for fMRI Performance Across TR Lengths.** To better understand how SGM performs across a variety of TR lengths (i.e., temporal resolutions), we systematically downsampled the TR for the above MICA dataset analysis (Figure S1). Using MATLAB's `resample` function, we resampled the natural 600ms TR in intervals of 200ms up to 2000ms, then performed SGM for fMRI parameter estimation in the same way as previous analyses. The main effect of downsampling TR was a slight reduction in the global time constant τ.

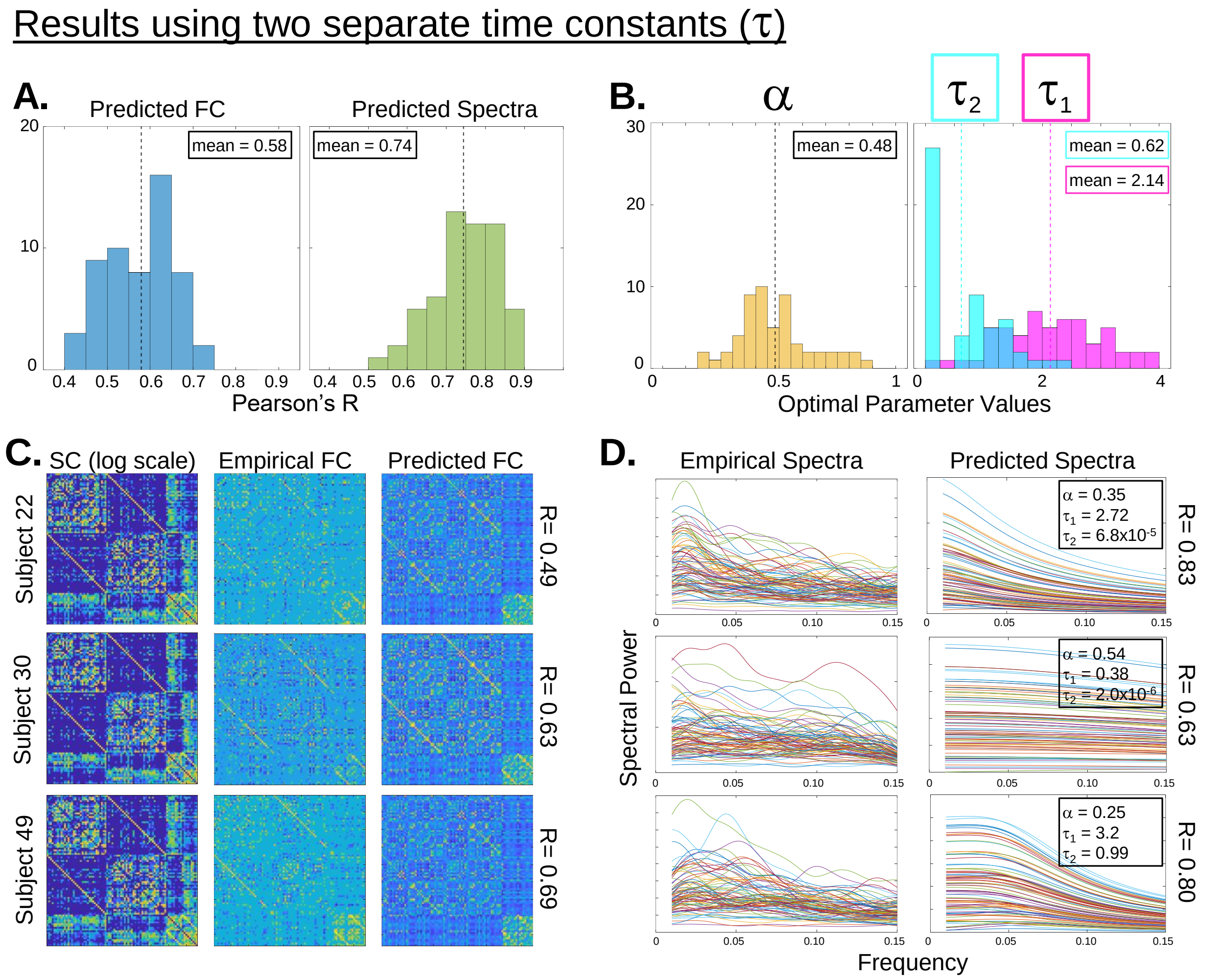

Figure S3: **Model fitting on UCSF data under the 3-parameter SGM.** We split the singular constant τ of the main model in Equation (7) into two: τ_1, the network diffusion time and τ_2, the cortical response time – contained in the alternate model Equation (8). Both τ_1 and τ_2 received identical range and initialization. The results of this new model are presented here closely following the main Figure 3. While the fitted values of τ_1 and τ_2 meaningfully diverged from each other and from the singular version τ, yet the end model was no better than the original, more parsimonious one.
